# The link between sleep quality and nutritional status in community-dwelling Iranian elderly

**DOI:** 10.1097/MD.0000000000042971

**Published:** 2025-06-27

**Authors:** Ali Kohanmoo, Asma Kazemi, Masoumeh Akhlaghi

**Affiliations:** a Department of Community Nutrition, School of Nutrition and Food Sciences, Shiraz University of Medical Sciences, Shiraz, Iran; b Nutrition Research Center, School of Nutrition and Food Sciences, Shiraz University of Medical Sciences, Shiraz, Iran.

**Keywords:** elderly, food intake, nutritional assessment, nutritional status, sleep quality

## Abstract

The prevalence of sleep deprivation is high among the elderly. Moreover, some evidence indicates interesting connections between sleep quality and nutritional factors. This study aimed to examine the relationship of nutritional status and sleep quality in Iranian elderly. A cross-sectional study was conducted on 305 community-dwelling elderly individuals aged ≥ 65 years. Sleep quality and nutritional status were assessed using the Pittsburgh Sleep Quality Index and the Mini Nutritional Assessment (MNA), respectively. Adjusted relationships were evaluated by logistic regression. Women had a higher prevalence of poor sleep quality than men (77.0% vs 48.9%), although both genders showed good overall nutritional status (89.1% for men and 85% for women). In men, there were significant associations between good sleep quality and food intake, protein food intake, and total MNA score in the crude model but not after adjustment for confounders. Women demonstrated significant associations between good sleep quality and intake of protein foods (OR = 0.23), dairy (OR = 0.30), fruit/vegetables (OR = 0.26), weight loss (OR = 0.25), and total MNA score (OR = 0.66) in the adjusted model. An association between sleep quality and nutritional status was observed only in women, which could be due to their poorer sleep quality compared to men. Given that the individuals were in relatively good nutritional status, future studies may assess these relationships in older adults with poorer nutritional status.

## 1. Introduction

Sleep is one of the most basic human needs.^[1]^ Sleep deprivation may have a wide range of adverse consequences, including cardiometabolic diseases, cognitive decline, impaired performance, and increased risk of accidents.^[1,2]^ The prevalence of sleep deprivation is high, especially among elderly. About 30% of community-dwelling older adults in the US get less than the recommended ≥ 7 hours sleep per day and 18% experience frequent insomnia or poor-quality sleep.^[3]^ The rates seem to be higher in Iran. A meta-analysis estimated the prevalence of sleep disorders among Iranian older adults to be 48.9%.^[4]^

The link between nutrition and sleep has been highlighted in the literature.^[5]^ As an example, foods rich in tryptophan, melatonin, and serotonin, such as protein foods and fruits, have shown to improve sleep quality.^[5]^ However, the relationship between nutritional status and sleep quality among elderly has been considered by few investigators. Yokoro et al reported that the diet variety score was lower in community-dwelling elderly Japanese women with sleep disturbance compared to normal sleepers.^[6]^ Also, Papadopoulou et al found that Greek elderly with better nutritional status had better sleep quality.^[7]^ Since the nutritional status strongly depends on sociocultural and economic characteristics of the population,^[8,9]^ investigating the relationship in different populations seems necessary.

In the current investigation, we evaluated nutritional status and sleep quality in a group of community-dwelling Iranian elderly. To assess nutritional status, we used the MNA which is a valid nutritional screening tool for detection of malnutrition in individuals aged ≥ 65.^[10]^ We hypothesized that sleep quality has a close relationship with MNA scores in Iranian elderly. Results of this study uncover possible relationships between sleep quality and overall nutritional status as well as components of nutrition and general health among elderly people in Iran.

## 2. Materials and methods

### 2.1. Study design

This cross-sectional study was conducted on 305 men and women aged ≥ 65 years in autumn and winter 2021 to 2022. The sample size was calculated based on results reported by other investigators^[11]^ who found a correlation coefficient of 0.241 for the association between sleep quality and nutritional status assessed by MNA tool, a power of 80%, and a type 1 error of 5%. The study was conducted according to the guidelines of the Declaration of Helsinki and all procedures involving human subjects and the protocol were approved by the Ethics Committee of Shiraz University of Medical Sciences with the approval code of IR.SUMS.SCHEANUT.REC.1400.002. All participants gave written informed consent after explanation of the study protocol.

### 2.2. Participants

Participants were collected from visitors of healthcare centers for elderly in Shiraz through simple cluster sampling. Three clusters were randomly selected out of 11 municipal areas in Shiraz. Subsequently, 1 center from each cluster was randomly chosen. Due to the restraints caused by COVID-19 pandemic, participants were recruited by convenience sampling in each center. Thus, all clients who attended the 3 centers from November 2021 to April 2022 were invited to participate in the study. Participants needed to be ≥ 65 years old and community-dwelling. Individuals who were afflicted with serious diseases such as cancer or cognitive disorders, or had been hospitalized during the past month were not included. General demographic information, medical history, medications, and smoking status were asked through interview. Physical activity was estimated as a confounding factor using a single-item questionnaire which has been validated before.^[12,13]^

### 2.3. Sleep quality

Sleep quality was assessed by the Pittsburgh Sleep Quality Index (PSQI) questionnaire. It assesses sleep quality and disturbances during the past month based on participant’s view.^[14]^ The questionnaire has been validated in elderly Iranian population.^[15]^ The PSQI contains items about sleep duration, sleep quality, sleep latency, sleep efficiency, sleep disturbances, sleeping medication, and daytime dysfunction. Each of these 7 components is scored from 0 to 3, with a total score of 0 to 21. Higher PSQI scores indicate poorer sleep quality. A cutoff point of < 6 is suggested as good sleep quality.^[14]^

### 2.4. Nutritional status

MNA questionnaire is a valid and reliable tool for assessing nutritional status in healthy adults^[16]^ and elderly with chronic diseases.^[17]^ MNA has also been validated for nutritional assessment of Iranian elderly.^[17]^ It is composed of 18 questions about food intake, weight loss, mobility, neuropsychological problems, body mass index, number of medications and meals, consumption of proteins, fruits and vegetables, and fluids, perceived nutritional and health status, and measurements of mid-arm and calf circumference.^[16]^ Total scores of 24 to 30, 17 to 23.5, and <17 points are categorized as normal nutritional status, at risk of malnutrition, and malnourished, respectively.

### 2.5. Statistical analysis

Statistical analyses were performed by SPSS version 16 (SPSS Inc., Chicago, IL). Demographic information was presented as number and percentage, and compared between men and women by chi-square test. In descriptive analysis of the inter-relationship between sleep quality and MNA components, MNA components were presented across gender-specific PSQI tertiles, and *P* values were calculated by Kruskal–Wallis test. The relationship of nutritional components of MNA and sleep quality was assessed by logistic regression in crude model and after adjustment for potential confounding factors (age, education, marital status, income level, loneliness, smoking, physical activity, sleeping pills, chronic pain, and self-reported psychological disorders). To compare results of logistic regression analysis between men and women, sex *P* value was considered when the sex was among the confounders in the adjusted regression model for all participants. The significance level was set at .05.

## 3. Results

Overall, 627 individuals were invited to participate in the study. Of these, 290 declined the invitation, and 32 did not meet the inclusion criteria. Finally, the study included 92 men and 213 women aged ≥ 65. There was no significant age difference between men and women; however, women had significantly lower education levels and lived alone more frequently than men. Additionally, women reported higher income levels and spent more on food than men (Table 1). The prevalence of poor sleep quality was significantly higher in women (48.9% in men and 77.0% in women; *P* < .001). Most participants had normal nutritional status, with no significant differences between genders, although women were slightly at higher risk of malnutrition (14.5% compared to 9.8%).

**Table 1:** General characteristics of the participants based on gender.*

	Men (n = 92)	Women (n = 213)	*P* value†
Age			
65–74.9 y	53 (57.6)	112 (52.6)	.713
75–84.9 y	23 (25.0)	61 (28.6)	
≥ 85 y	16 (17.4)	40 (18.8)	
Education			
School	71 (77.2)	188 (88.3)	.013
University	21 (22.8)	25 (11.7)	
Marital status			
Single	10 (10.9)	85 (39.9)	<.001
Married	82 (89.1)	128 (60.1)	
Living situation			
Alone	4 (4.3)	47 (22.1)	<.001
With family	88 (95.7)	166 (77.9)	
Family income			
<10 M.R.	7 (7.6)	17 (8.0)	<.001
10–20 M.R.	60 (65.2)	84 (39.4)	
20–40 M.R.	22 (23.9)	77 (36.2)	
>40 M.R.	3 (3.3)	35 (16.4)	
Family food expenses			
<0.5 M.R.	9 (9.8)	15 (7.0)	.001
0.5–1 M.R.	59 (64.1)	91 (42.7)	
1–2 M.R.	21 (22.8)	75 (35.2)	
>2 M.R.	3 (3.3)	32 (15.0)	
Sleep quality			
Good (≤5)	47 (51.1)	49 (23.0)	<.001
Poor (>5)	45 (48.9)	164 (77.0)	
MNA‡			
Malnourished	1 (1.1)	1 (0.5)	
At risk of malnutrition	9 (9.8)	31 (14.5)	.381
Normal nutritional status	82 (89.1)	181 (85.0)	

MNA = Mini Nutritional Assessment.

* Data are expressed as number and percentage.

† *P* value was obtained by chi-square test.

‡ Scores of < 17, 17 to 23.5, and 24 to 30 points were categorized as malnourished, at risk of malnutrition, and normal nutritional status, respectively.

For a trend view, scores of MNA components were expressed across gender-specific PSQI tertiles (Table 2). Both genders showed a significant relationship between MNA total score and PSQI tertiles. In men, the intake of protein foods (dairy, legumes/eggs, and meats) and self-view of nutritional status were the only MNA components which decreased across PSQI tertiles. In women, scores of declined food intake and weight loss, psychological stress/acute disease, taking > 3 prescribed drugs, self-feeding, and intake of protein foods and fruit/vegetables were MNA components that significantly decreased across PSQI tertiles. Intake of fluids increased from tertile 1 to tertile 3 of PSQI in women.

**Table 2:** Inter-relationship between scores of MNA components and sleep quality.*^,^†

Scores of MNA components ↓	Gender-specific PSQI tertiles							
	Men				Women			
	Tertile 1 (n = 20)	Tertile 2 (n = 45)	Tertile 3 (n = 27)	*P* value‡	Tertile 1 (n = 64)	Tertile 2 (n = 85)	Tertile 3 (n = 64)	*P* value‡
Declined food intake during past 3 months	1.85 ± 0.37	1.89 ± 0.32	1.70 ± 0.54	.237	1.91 ± 0.28	1.82 ± 0.44	1.73 ± 0.48	.034
Weight loss during past 3 months	2.65 ± 0.75	2.51 ± 0.84	2.41 ± 0.89	.522	2.84 ± 0.41	2.46 ± 0.84	2.38 ± 0.92	.001
Mobility	2.00 ± 0	2.00 ± 0	2.00 ± 0	1	2.00 ± 0	1.99 ± 0.05	1.99 ± 0.06	.631
Psychological stress/acute disease	1.85 ± 0.37	1.78 ± 0.46	1.69 ± 0.54	.491	1.83 ± 0.42	1.62 ± 0.67	1.42 ± 0.70	.001
Neuropsychological problems	1.90 ± 0.31	1.72 ± 0.49	1.65 ± 0.59	.232	1.83 ± 0.48	1.79 ± 0.42	1.73 ± 0.44	.172
Low body mass index	2.40 ± 1.10	2.80 ± 0.50	2.70 ± 0.72	.324	2.80 ± 0.57	2.85 ± 0.45	2.97 ± 0.18	.082
Independent living	1.00 ± 0	1.00 ± 0	1.00 ± 0	1	1.00 ± 0	1.00 ± 0	1.00 ± 0	1
Taking > 3 prescribed drugs	0.80 ± 0.41	0.73 ± 0.45	0.52 ± 0.51	.077	0.61 ± 0.49	0.35 ± 0.48	0.25 ± 0.44	<.001
Pressure ulcers	1.00 ± 0	1.00 ± 0	1.00 ± 0	1	1.00 ± 0	1.00 ± 0	1.00 ± 0	1
Meal frequency	1.98 ± 0.11	1.93 ± 0.25	1.78 ± 0.51	.167	1.86 ± 0.35	1.91 ± 0.29	1.85 ± 0.35	.504
Protein food intake	0.70 ± 0.34	0.63 ± 0.34	0.41 ± 0.34	.009	0.65 ± 0.35	0.44 ± 0.37	0.45 ± 0.38	.001
Dairy products	0.80 ± 0.41	0.67 ± 0.48	0.65 ± 0.48	.463	0.86 ± 0.35	0.63 ± 0.48	0.60 ± 0.49	.002
Legumes/eggs	0.85 ± 0.37	0.89 ± 0.32	0.70 ± 0.47	.130	0.85 ± 0.35	0.72 ± 0.45	0.77 ± 0.42	.145
Meats	0.75 ± 0.44	0.71 ± 0.46	0.48 ± 0.49	.064	0.59 ± 0.50	0.55 ± 0.49	0.45 ± 0.50	.219
Fruit/vegetable intake	0.80 ± 0.38	0.77 ± 0.36	0.67 ± 0.39	.310	0.86 ± 0.31	0.73 ± 0.40	0.64 ± 0.41	.003
Fluids	0.90 ± 0.21	0.96 ± 0.18	0.93 ± 0.18	.297	0.89 ± 0.24	0.84 ± 0.26	0.94 ± 0.21	.009
Self-feeding	1.83 ± 0.44	1.91 ± 0.27	1.74 ± 0.40	.074	1.99 ± 0.06	1.87 ± 0.32	1.88 ± 0.32	.018
Self-view of nutritional status	1.95 ± 0.22	1.93 ± 0.25	1.74 ± 0.47	.030	1.92 ± 0.27	1.82 ± 0.38	1.80 ± 0.39	.107
Self-view of health status	1.60 ± 0.50	1.66 ± 0.50	1.41 ± 0.69	.335	1.51 ± 0.57	1.44 ± 0.64	1.27 ± 0.69	.126
Mid-arm circumference score	1.00 ± 0	1.00 ± 0	0.96 ± 0.19	.300	1.00 ± 0	1.00 ± 0	0.99 ± 0.06	.312
Calf circumference score	1.00 ± 0	1.00 ± 0	0.96 ± 0.19	.300	0.95 ± 0.21	0.95 ± 0.21	0.97 ± 0.18	.873
Total MNA score	27.20 ± 2.19	27.22 ± 1.72	25.26 ± 3.65	.018	27.45 ± 1.96	25.88 ± 2.29	25.25 ± 2.35	<.001

MNA = Mini Nutritional Assessment, PSQI = Pittsburgh Sleep Quality Index.

* Values are means ± SD.

† Sleep quality was assessed by PSQI which scores sleep quality in reverse mode.

‡ *P* values were calculated by Kruskal–Wallis test.

The relationships between sleep quality and food-related components of MNA were examined by logistic regression analysis (Table 3). Men showed significant associations between good sleep quality and the scores of protein food intake, declined food intake, and total MNA in the crude model. However, after adjustment for potential confounders these associations disappeared. Women demonstrated significant associations between good sleep quality and the intake of protein foods (OR = 0.27; 95% CI: 0.11–0.66), dairy (OR = 0.29, 95% CI: 0.12–0.70), and fruit/vegetables (OR = 0.23; 95% CI: 0.08–0.67), and also the scores of declined food intake (OR = 0.21; 95% CI: 0.06–0.81), weight loss (OR = 0.22; 95% CI: 0.09–0.55), and total MNA (OR = 0.62; 95% CI: 0.51–0.75) in the crude model. Except for declined food intake, all these components remained significant in the adjusted model. Sex *P* values revealed significant differences between men and women in the MNA components.

**Table 3:** Logistic regression analysis for the relationship between scores of MNA components and poor sleep quality* in men and women.

	Men				Women				Sex *P* value
	Crude OR (95% CI)	*P* value	Adjusted OR (95% CI)†	*P* value	Crude OR (95% CI)	*P* value	Adjusted OR (95% CI)†	*P* value	
Protein foods	0.27 (0.08, 0.91)	.035	0.49 (0.11, 2.24)	.356	0.27 (0.11, 0.66)	.004	0.23 (0.08, 0.68)	.008	.001; 3.28 (1.61, 6.70)
Meats	0.42 (0.17, 1.02)	.060	0.46 (0.15, 1.43)	.179	0.80 (0.42, 1.54)	.510	0.78 (0.36, 1.69)	.531	.001; 3.28 (1.62, 6.63)
Dairy products	0.89 (0.37, 2.17)	.799	1.52 (0.46, 5.00)	.492	0.29 (0.12, 0.70)	.005	0.30 (0.11, 0.78)	.014	.001; 3.46 (1.70, 7.02)
Fruit/vegetables	0.62 (0.21, 1.88)	.401	1.62 (0.37, 7.21)	.524	0.23 (0.08, 0.67)	.007	0.26 (0.08, 0.85)	.025	.001; 3.45 (1.70, 7.03)
Fluids	3.55 (0.32, 38.9)	.299	44.1 (0.32, 70.9)	.132	1.12 (0.31, 4.08)	.868	1.17 (0.25, 5.53)	.842	.001; 3.44 (1.70, 7.00)
Declined food intake	0.29 (0.09, 0.96)	.043	0.29 (0.06, 1.35)	.114	0.21 (0.06, 0.81)	.023	0.25 (0.05, 1.22)	.086	.001; 3.48 (1.71, 7.10)
Weight loss	0.77 (0.46, 1.29)	.320	0.72 (0.38, 1.38)	.323	0.22 (0.09, 0.55)	.001	0.25 (0.09, 0.69)	.007	<.001; 3.59 (1.76, 7.33)
MNA total score	0.73 (0.59, 0.91)	.005	0.83 (0.62, 1.12)	.221	0.62 (0.51, 0.75)	<.001	0.66 (0.52, 0.84)	.001	.001; 3.26 (1.58, 6.72)

MNA = Mini Nutritional Assessment.

* Poor sleep quality determined by PSQI score > 5 was used in the analysis as dependent variable.

† Multivariable model adjusted for age, education, marital status, income level, living condition (alone or with family), smoking, physical activity, the use of sleeping pills, chronic pain, and self-reported psychological disorders.

## 4. Discussion

This was a cross-sectional study in a group of apparently healthy elderly. The study showed high prevalence of poor sleep quality among both genders, especially women. Overall prevalence was 68.5%, and women had substantially higher rates. Investigators in other parts of the world have also shown high prevalence of poor sleep quality among elderly and the rates are generally higher among women compared to men.^[18,19]^ Additionally, women showed positive associations between sleep quality and items of MNA questionnaire that related to food intake, but such associations were not observed in men after controlling potential confounders.

### 4.1. Dairy and sleep quality

The consumption of dairy products decreased across tertiles of poor sleep quality in women. Also, in regression analysis there was a positive relationship between dairy intake and sleep quality in women. Although with uncertainty, the relationship of dairy products and sleep quality has been suggested in previous investigations.^[20]^ For instance, in a cross-sectional study on 270 university students, consumption of healthy dairy including milk, cottage cheese, and yogurt, but not unhealthy dairy such as cheese and processed cheese, was predictive of sleep quality.^[20]^

Dairy products are rich in protein and the amino acid tryptophan, which is the precursor of serotonin and then melatonin, a hormone responsible for the sleep–wake cycle.^[21]^ In a randomized controlled trial, the intake of lactalbumin, which is one of the important proteins of milk, increased concentration of tryptophan before bedtime and reduced sleepiness and improved attention the following morning.^[22]^ Also, a systematic review of epidemiologic and interventional studies suggested that consumption of milk and dairy products in the context of a balanced diet may be effective in improving sleep quality.^[23]^

### 4.2. Fruits/vegetables intake and sleep quality

Women had a decreasing trend in consumption of fruits/vegetables across tertiles of poor sleep quality. They also indicated an association between fruits/vegetables intake and sleep quality in regression analysis. In agreement, a number of cross-sectional studies have suggested the positive link between fruits and vegetables intake and sleep quality. For instance, a cross-sectional study with 21,027 university students from 28 countries showed an inverse association between fruits and vegetables consumption and poor sleep quality.^[24]^ Moreover, a 3-month online program that increased fruits and vegetables intake of 1165 adults improved sleep quality and insomnia symptoms in women.^[25]^

The relationship between fruits/vegetables consumption and sleep is said to be reciprocal, meaning that both poor sleep and low fruits/vegetables intake can promote each other.^[26]^ For instance, poor sleep may increase desire for eating energy-dense foods and subsequently decrease fruits/vegetables intake by disrupting the appetite-regulating hormones leptin and ghrelin. Conversely, consumption of fruits and vegetables may improve sleep through their antioxidant and anti-inflammatory properties.^[27]^

### 4.3. Other diet components and sleep quality

Other food-related components of MNA questionnaire did not show significant relationships with sleep quality in either genders. Legumes/eggs, meats, and fluids did not indicate a significant trend across PSQI tertiles, nor did they show a relationship in the regression analysis (data not shown). Studies from other investigators have shown more or less similar results. For instance, an RCT on healthy adults did not show an effect from mild dehydration on sleep quality.^[28]^ Another RCT on healthy adults did not find a significant effect from a meal containing animal proteins on sleep quality.^[29]^ Contrarily, data of a cohort on elderly showed that compared to individuals in the lowest tertile of meat consumption, those in the highest tertile showed increased incidence of poor sleep quality during the 3-year follow-up.^[30]^ The reason for the lack of beneficial effect of meats and eggs on sleep despite their high protein content could be the high amount of large neutral amino acids in such foods. This may reduce the supply of tryptophan and tyrosine to the brain, which leads to reduction in the synthesis of the neurotransmitters involved in sleep regulation.^[31]^

### 4.4. Weight loss and sleep quality

Participants of this study were in good nutritional status. Only 0.7% had malnutrition, and 13.1% were at risk of malnutrition. Moreover, the majority of them had full score for mid-arm and calf circumference, which also indicates that they mostly were not malnourished. However, even in such apparently nutritionally healthy population, some individuals had weight loss and declined food intake in the past 3 months which could be indicative of a risk for future malnutrition.^[32]^ Weight loss or declined food intake had a positive relationship with poor sleep quality in women. In agreement with this finding, Liu et al reported that weight loss was negatively associated with sleep quality in Hainanese centenarians.^[33]^ The evidence for the relationship of sleep and nutritional status^[7,34]^ or malnutrition^[35,36]^ is more robust. In this regard, Papadopoulou et al reported that in a community-dwelling population in Greece, a better nutritional status was associated with better sleep quality.^[7]^ Similar results were found by Zhao et al in community-dwelling older adults in China.^[35]^ These results suggest that poor sleep quality may trigger declined nutritional status in the elderly, or, declined nutritional status may promote poor sleep quality.

### 4.5. The role of gender

Men and women did not significantly differ in the MNA score. But women had significantly poorer sleep quality and showed a relationship between total MNA score and sleep quality. Also, relationships existed for some of the MNA components such as protein foods, dairy, fruit/vegetables, and weight loss only in women. Previous studies are mostly in agreement with the gender difference found here. For instance, good sleep quality was associated with a dietary pattern high in fruits/vegetables and dairy products in female police officers, but not in males.^[37]^ Also, in a 3-month longitudinal study, increased fruit/vegetables intake by 3 servings improved insomnia and sleep quality only in women.^[25]^ Contrarily, some investigators have reported associations of poor sleep and low fruit/vegetables intake in men, but not in women.^[38,39]^ The reasons for gender differences are not clear but higher rates of poor sleep quality in women may allow consuming specific dietary items to exert their beneficial effects on sleep. In addition, women have generally higher intention for fruits and vegetables consumption, and this may improve their sleep quality. On the other hand, because female workers may be physically and mentally affected by workload conditions and also housework, they may not get enough time to take care of their diet. This could be the reason for the lack of associations for women in some investigations.^[39,40]^

### 4.6. Strengths and limitations

The study had strengths and limitations. Components of MNA were individually assessed to find the components that may have a relationship with sleep quality. Also, separating the analysis by gender helped to explore the relationships specifically in each gender. However, participants were relatively in good nutritional status. Therefore, the results cannot be generalized to malnourished elderly groups or those with poor nutritional conditions. Lastly, the cross-sectional design of the study did not allow discovering cause-effect relationships.

### 4.7. Conclusions

Overall, results of this study showed high prevalence of poor sleep quality in the participants, especially women. Women showed positive associations between sleep quality and intake of protein foods, dairy, and fruit/vegetables, and a negative association between sleep quality and weight loss during the last 3 months. Such associations were not observed among men which could be due to their better sleep quality. Sleep and nutrition are believed to have a bi-directional relationship. The cross-sectional design of the study did not allow to determine which problem preceded or contributed to the occurrence of the other. High-quality longitudinal studies or well-controlled trials are needed to clarify this issue. Healthcare efforts that help improve either one may work to improve overall health. Another point is that the observation of significant relationships between MNA components and sleep quality in elderly participants with good nutritional status is an interesting finding which needs to be considered in clinical settings. However, results need to be tested in elderly populations with poorer nutrition.

## Author contributions

**Conceptualization:** Ali Kohanmoo, Masoumeh Akhlaghi.

**Data curation:** Ali Kohanmoo.

**Formal analysis:** Ali Kohanmoo, Asma Kazemi, Masoumeh Akhlaghi.

**Funding acquisition:** Masoumeh Akhlaghi.

**Investigation:** Ali Kohanmoo.

**Project administration:** Masoumeh Akhlaghi.

**Software:** Ali Kohanmoo.

**Supervision:** Asma Kazemi.

**Writing – original draft:** Ali Kohanmoo, Masoumeh Akhlaghi.

**Writing – review & editing:** Asma Kazemi, Masoumeh Akhlaghi.
